# Supplementary material for: HIVprotI: an integrated web based platform for prediction and design of HIV proteins inhibitors
Source: J Cheminform. 2018 Mar 9;10:12. doi: 10.1186/s13321-018-0266-y (PMC5845081; doi:10.1186/s13321-018-0266-y)
Supplement: Supplementary file 2 — Additional file 2. Source code of HIVProtI web server. [file 13321_2018_266_MOESM2_ESM.zip › HIVprotI_Source-code/hivprotI/weka-3-6-6/doc/weka/classifiers/functions/pace/MixtureDistribution.html]

MixtureDistribution


---


|  |  |  |
| --- | --- | --- |
| **Overview** \| **Package** \| **Class** \| **Tree** \| **Deprecated** \| **Index** \| **Help** \| **Weka's home** | |  |
| **PREV CLASS**   **NEXT CLASS** | **FRAMES**    **NO FRAMES**     **All Classes** |  |
| SUMMARY: NESTED \| FIELD \| CONSTR \| METHOD | DETAIL: FIELD \| CONSTR \| METHOD |  |


---


## weka.classifiers.functions.pace Class MixtureDistribution

```
java.lang.Object
  weka.classifiers.functions.pace.MixtureDistribution
```

**All Implemented Interfaces:**: RevisionHandler, TechnicalInformationHandler

**Direct Known Subclasses:**: ChisqMixture, NormalMixture

---

``` public abstract class MixtureDistribution extends java.lang.Object implements TechnicalInformationHandler, RevisionHandler ```

Abtract class for manipulating mixture distributions.

REFERENCES

Wang, Y. (2000). "A new approach to fitting linear models in high
dimensional spaces." PhD Thesis. Department of Computer Science,
University of Waikato, New Zealand.

Wang, Y. and Witten, I. H. (2002). "Modeling for optimal probability
prediction." Proceedings of ICML'2002. Sydney.

**Version:**
:   $Revision: 1.5 $

**Author:**
:   Yong Wang (yongwang@cs.waikato.ac.nz)

---

| **Field Summary** | |
| --- | --- |
| `static int` | `NNMMethod`             The nonnegative-measure-based method |
| `static int` | `PMMethod`             The probability-measure-based method |


**Constructor Summary**

`MixtureDistribution()`


| **Method Summary** | |
| --- | --- |
| `PaceMatrix` | `empiricalProbability(DoubleVector data, PaceMatrix intervals)`             Computes the empirical probabilities of the data over a set of intervals. |
| `void` | `fit(DoubleVector data)`             Fits the mixture (or mixing) distribution to the data. |
| `void` | `fit(DoubleVector data, int method)`             Fits the mixture (or mixing) distribution to the data. |
| `DiscreteFunction` | `fitForSingleCluster(DoubleVector data, int method)`             Fits the mixture (or mixing) distribution to the data. |
| `abstract  PaceMatrix` | `fittingIntervals(DoubleVector data)`             Contructs the set of fitting intervals for mixture estimation. |
| `DiscreteFunction` | `getMixingDistribution()`             Gets the mixing distribution |
| `TechnicalInformation` | `getTechnicalInformation()`             Returns an instance of a TechnicalInformation object, containing detailed information about the technical background of this class, e.g., paper reference or book this class is based on. |
| `abstract  PaceMatrix` | `probabilityMatrix(DoubleVector s, PaceMatrix intervals)`             Contructs the probability matrix for mixture estimation, given a set of support points and a set of intervals. |
| `abstract  boolean` | `separable(DoubleVector data, int i0, int i1, double x)`             Return true if a value can be considered for mixture estimatino separately from the data indexed between i0 and i1 |
| `void` | `setMixingDistribution(DiscreteFunction d)`             Sets the mixing distribution |
| `abstract  DoubleVector` | `supportPoints(DoubleVector data, int ne)`             Contructs the set of support points for mixture estimation. |
| `java.lang.String` | `toString()`             Converts to a string |

**Methods inherited from class java.lang.Object**

`equals, getClass, hashCode, notify, notifyAll, wait, wait, wait`

**Methods inherited from interface weka.core.RevisionHandler**

`getRevision`

**Field Detail**

### NNMMethod

```
public static final int NNMMethod
```

:   The nonnegative-measure-based method

    **See Also:**: Constant Field Values

---


### PMMethod

```
public static final int PMMethod
```

:   The probability-measure-based method

    **See Also:**: Constant Field Values


**Constructor Detail**

### MixtureDistribution

```
public MixtureDistribution()
```


**Method Detail**

### getTechnicalInformation

```
public TechnicalInformation getTechnicalInformation()
```

:   Returns an instance of a TechnicalInformation object, containing
    detailed information about the technical background of this class,
    e.g., paper reference or book this class is based on.

    :   **Specified by:**: `getTechnicalInformation` in interface `TechnicalInformationHandler`
    :   **Returns:**: the technical information about this class

---


### getMixingDistribution

```
public DiscreteFunction getMixingDistribution()
```

:   Gets the mixing distribution

    :   **Returns:**: the mixing distribution

---


### setMixingDistribution

```
public void setMixingDistribution(DiscreteFunction d)
```

:   Sets the mixing distribution

    :   **Parameters:**: `d` - the mixing distribution

---


### fit

```
public void fit(DoubleVector data)
```

:   Fits the mixture (or mixing) distribution to the data. The default
    method is the nonnegative-measure-based method.

    :   **Parameters:**: `data` - the data, supposedly generated from the mixture model

---


### fit

```
public void fit(DoubleVector data,
                int method)
```

:   Fits the mixture (or mixing) distribution to the data.

    :   **Parameters:**: `data` - the data supposedly generated from the mixture: `method` - the method to be used. Refer to the static final variables of this class.

---


### fitForSingleCluster

```
public DiscreteFunction fitForSingleCluster(DoubleVector data,
                                            int method)
```

:   Fits the mixture (or mixing) distribution to the data. The data is
    not pre-clustered for computational efficiency.

    :   **Parameters:**: `data` - the data supposedly generated from the mixture: `method` - the method to be used. Refer to the static final variables of this class. **Returns:**: the generated distribution

---


### separable

```
public abstract boolean separable(DoubleVector data,
                                  int i0,
                                  int i1,
                                  double x)
```

:   Return true if a value can be considered for mixture estimatino
    separately from the data indexed between i0 and i1

    :   **Parameters:**: `data` - the data supposedly generated from the mixture: `i0` - the index of the first element in the group: `i1` - the index of the last element in the group: `x` - the value **Returns:**: true if a value can be considered

---


### supportPoints

```
public abstract DoubleVector supportPoints(DoubleVector data,
                                           int ne)
```

:   Contructs the set of support points for mixture estimation.

    :   **Parameters:**: `data` - the data supposedly generated from the mixture: `ne` - the number of extra data that are suppposedly discarded earlier and not passed into here **Returns:**: the set of support points

---


### fittingIntervals

```
public abstract PaceMatrix fittingIntervals(DoubleVector data)
```

:   Contructs the set of fitting intervals for mixture estimation.

    :   **Parameters:**: `data` - the data supposedly generated from the mixture **Returns:**: the set of fitting intervals

---


### probabilityMatrix

```
public abstract PaceMatrix probabilityMatrix(DoubleVector s,
                                             PaceMatrix intervals)
```

:   Contructs the probability matrix for mixture estimation, given a set
    of support points and a set of intervals.

    :   **Parameters:**: `s` - the set of support points: `intervals` - the intervals **Returns:**: the probability matrix

---


### empiricalProbability

```
public PaceMatrix empiricalProbability(DoubleVector data,
                                       PaceMatrix intervals)
```

:   Computes the empirical probabilities of the data over a set of
    intervals.

    :   **Parameters:**: `data` - the data: `intervals` - the intervals **Returns:**: the empirical probabilities

---


### toString

```
public java.lang.String toString()
```

:   Converts to a string

    :   **Overrides:**: `toString` in class `java.lang.Object`
    :   **Returns:**: a string representation


---


|  |  |  |
| --- | --- | --- |
| **Overview** \| **Package** \| **Class** \| **Tree** \| **Deprecated** \| **Index** \| **Help** \| **Weka's home** | |  |
| **PREV CLASS**   **NEXT CLASS** | **FRAMES**    **NO FRAMES**     **All Classes** |  |
| SUMMARY: NESTED \| FIELD \| CONSTR \| METHOD | DETAIL: FIELD \| CONSTR \| METHOD |  |


---
